# Psychometric Assessment of an Online Self-Test Measuring Risky Eating Behavior, Depression, Social Anxiety, and Self-Injury in Mexican Adolescents

**DOI:** 10.3390/ijerph20010399

**Published:** 2022-12-27

**Authors:** Gilda Gómez-Peresmitré, Romana Silvia Platas-Acevedo, Rodrigo León-Hernández, Gisela Pineda-García, Rebeca Guzmán-Saldaña

**Affiliations:** 1 Faculty of Psychology, The National Autonomous University of Mexico, Av. Universidad 3004 Col Copilco-Universidad, Alcaldía Coyoacán, Mexico City C.P. 04510, Mexico; 2 National Council of Science and Technology, Avenida Insurgentes Sur 1582, Crédito Constructor, Ciudad de México C.P. 03940, Mexico; 3 Faculty of Medicine and Psychology, Autonomous University of Baja California, Cal. University 14418, International Industrial Park, Tijuana C.P. 22390, Mexico; 4 Institute of Health Sciences, Autonomous University of the State of Hidalgo, Camino a Tilcuautla s/n Pueblo San Juan Tilcuautla, Hidalgo C.P. 42160, Mexico

**Keywords:** validity and reliability, online test, self-detection, prevention of risk factors, eating disorders

## Abstract

The objective of this study was to evaluate the validity and reliability of OTESSED, an online test for the self-detection and prevention of risk factors for eating disorders and related problems, such as depression, social anxiety, and self-injury, in samples of male and female adolescents. Participants formed a non-probability sample of N = 577 high school students. The mean ages of boys and girls were the same (Mage = 15.61; SD = 0.73). Among the main results, scales of depression, self-injury, social anxiety, and risky eating behavior (REB) with construct validity (CFA) were obtained. The first two showed the same structure (two factors per sex), with an appropriate reliability omega value (0.92), and a similar percentage of explained variance (≥50). The REB scale presented two factors for boys and three for girls, with an appropriate omega value (0.88) and explained variance percentage (0.56). The instrument validation process was completed, meeting the discriminant validity criterion for each scale of the OTESSED.

## 1. Introduction

Adolescence is a stage in the transition to adulthood with high biological, psychological, and social risks. Adolescents have just finished experiencing the changes of puberty when entering this new stage of significant challenges, including alcohol or drug use, sexual activity, and violence, as a means of striving for independence. The transition can lead to a lack of emotional control or to experiencing new emotions such as depression/anxiety about physical development, relationships with others, and oneself. However, severe mental health conditions also emerge during adolescence: risk-taking behaviors and limit testing increase in this age group. In addition, young teenagers learn to establish boundaries and limitations [1,2]. 

According to the World Health Organization (WHO) [3], any person between the ages of 10 and 19 is an adolescent and is part of what the same organization defines as “young people,” to refer to individuals between the ages of 10 and 24. In early adolescence (ages 10 to 14), children look for close relationships with friends, experience a changing body, act out, and engage in risk-taking behavior. Middle adolescence (ages 15 to 17) is characterized by increased importance on intimate relationships, physical appearance, and trying to achieve independence from parents. Finally, from ages 18 to 21, late adolescence is characterized by a smaller, more intimate peer group, intimate relationships, and a closer relationship with parents [2,4,5].

It must be well established that the theoretical models that have received the most empirical support are based on the assumption that social anxiety, depression, and eating disorders are characterized by a shared etiology [6]. Anxiety disorders and depression are among the most prevalent mental disorders in adolescence. Both are the most common comorbid diagnoses of eating disorders, especially in adolescence. In a recent study of women aged 15 to 25 years, those who had experienced a lifetime major depressive disorder or anxiety disorder were four times more likely to have an eating disorder [6,7]. Anxiety and depression are the most frequent emotional disorders that affect an individual’s social, personal, and academic–occupational areas and are closely related to self-harm and suicidal ideation [8]. Although more than two-thirds of suicide deaths in adolescence/young adulthood have occurred with no previous suicidal behavior or previous self-injurious thoughts and behaviors, adolescents have a much higher risk of dying by suicide than previously reported in this age group [8,9]. 

The American Psychiatric Association (APA), in the Diagnostic Statistical Manual (DSM-5) [10], points to anxiety as a state of excessive worry about a wide range of events or activities that lasts for more than six months. The individual suffering from anxiety finds it difficult to control this constant state of worry, including symptoms such as restlessness and impatience, early fatigue, difficulty concentrating or having a blank mind, irritability, muscle tension, and sleep disturbances.

Anxiety disorders may present as panic attacks or excessive worry. Roughly 3.6% of adolescents aged 10–14 years and 4.6% of those aged 15–19 years suffer from an anxiety disorder, while 1.1% of adolescents aged 10–14 years and 2.8% of those aged 15–19 years suffer from depression [11]. Depression and anxiety have some of the same symptoms, such as rapid and unexpected changes in mood [12]. However, what best characterizes the relationship of these disorders is the negative relationship between age and depression, social anxiety, and disorders in eating behavior. Younger age appeared to augment the association of anxiety/depression and eating disorder symptomatology [13]. Social withdrawal is one of the consequences of a depressed mood, which can increase feelings of sadness, loneliness, and isolation. Comorbid social anxiety hinders recovery efforts, as avoidance of interpersonal situations and fear of negative evaluations interfere with participation in treatment and the building of a good therapeutic relationship. In adolescent girls and young adults, comorbid anxiety disorders are associated with increased eating disorder psychopathology [6]. Depression, on the other hand, can lead to suicide. Suicidal risk is the likelihood of suicide based on a subject’s previous and current experience [14]. The importance of detecting youth at suicidal risk is due to the probability of decreasing, and ideally avoiding, such behavior because there is evidence, and studies, showing that children with suicidal ideation are more likely to commit suicide than those without it [15,16]. In addition, the life histories of children with suicidal ideation can provide valuable information about the risk factors that may lead to such behavior.

However, one of the problems that cause the most concern is self-injury. Psychological problems in adolescence are the leading cause of morbidity and disability during this period, with suicide being the third most common cause of mortality among adolescents (World Health Organization) [17]. 

Concerning suicidal thinking in Mexican adolescents, it stands out that 6.9% of the adolescent population has had some suicidal thoughts in their lifetime, a figure significantly higher than the 5.1% reported in the ENSANUT (2018–19) (acronym in Spanish, National Health and Nutrition Survey) [18]. Although no significant increases were found when disaggregated by sex, females reported a higher prevalence of this type of thought than males (8.8% and 5.1%, respectively). Further, 6% of the adolescent population said that they had harmed themselves to take their own life, a figure significantly higher than the 3.9% reported in the ENSANUT 2018–19. This behavior was reported in 10% of females, a figure statistically higher than the 6.1% reported in the ENSANUT 2018–19 [18].

Recently, in Mexico, the ENSANUT 2021 [19] indicated that 6.9% of adolescents had thoughts of suicide, while the Ministry of Health [20] reported that between 600 and 700 adolescents seek consultation for the first time for problems with depression and social anxiety, accompanied by risky eating behaviors, self-injury, and suicide attempts. The REB Scale (risky eating behavior) [21,22,23], the Depression Scale [14,24,25], and the Social Anxiety Scale [26,27,28] are self-report instruments tested in the Mexican adolescent population. They have been validated in Mexico and different countries, allowing early detection of the emotional problems faced by adolescents.

Although there are known and effective treatments for mental disorders, more than 75% of affected individuals in low- and middle-income countries receive no treatment [29]. Barriers to effective care include a lack of resources and trained healthcare providers, and the stigma associated with mental disorders [11]. 

It remains to be noted that adolescents can also become trapped with eating and body image problems, with serious health repercussions. They become vulnerable to negative pressures on body image and weight, leading to disordered eating behaviors. In addition, the sociocultural pressure derived from esthetic ideals or beauty stereotypes that adolescents perceive to violate their perception of and satisfaction with their body image [30,31]. Dissatisfaction with body image is the discomfort that a person feels towards their body; it is directly related to the need for people to modify their bodies through extreme or restrictive diets or purgative or compensatory behaviors [32].

These variables, satisfaction/dissatisfaction with body image, have been the most researched. It is known that women are more dissatisfied than men because they overestimate their body weight regardless of their actual weight. Even if they are thin, they always want to be thinner (among women, the expected thinness is never reached). On the other hand, dissatisfaction is also found among men, because they want to be more muscular and thicker, not fat (among men, the desired muscle is never reached). In this response of satisfaction/dissatisfaction, there is another variable called alteration of body image. It can be produced in the form of overestimation or underestimation. In women’s dissatisfaction, it has been found that overestimation is present. At the same time, in that of men, underestimation is present. The first is the case of anorexia nervosa, and the second is muscular dysmorphic disorder (bigorexia) [33,34,35,36]. 

The media are the most potent promoters of the thin body esthetic model, which is unrealistic for Western society, generating a practically unattainable model through healthy means [36,37,38]. For decades, the media flooded television and social networks with images of idealized, drastically thin figures, promoting them in advertising campaigns on the street, public transport, and other public places.

Advertisements show skinny and toned models, mostly edited with computerized programs. With the help of technology, they are edited and altered, creating an unrealistic and unattainable standard. Some researchers [37,38,39,40] have provided evidence of the relationship between exposure to such ideal images of thinness and increased body dissatisfaction among adolescent girls and young women, particularly those with pre-existing body image problems [41].

One of the social networks with the most significant popularity among young people is Instagram. Wells et al. [42] pointed out that the images disseminated on social networks worsen body image problems for one in three adolescents. Likewise, [43,44] conducted systematic reviews and meta-analyses showing that social network use is associated with body image concerns and eating disorders. The photos disseminated on social networks feature photos and selfies posted by actors, singers, and famous people of the moment, including schoolmates. People who edit their photos and videos may use specific lighting strategies, camera angles, makeup, and photo filters that enhance their appearance to present their most attractive versions [37,38]. 

Among the obstacles and consequent recommendations for better and earlier use of mental health services, reference is made to the time lag that occurs between the gestation of most mental disorders [45] that occurs in adolescence (the peak age being 11 years) [23,46,47] and the diagnosis and treatment of these disorders that occurs in adulthood. Knowing the causes of this mismatch and seeking to eliminate it may be objectives to achieve in order to contribute to the well-being and improve the quality of life of an essential population of young people [48,49]. It has also been suggested that it is crucial to contribute to “awareness,” i.e., that young people become aware ideally of the presence of risk factors (or symptoms and mental health disorders) for a diagnosis, and that the earlier a diagnosis is made, the better. It is required, among many other things, to develop valid and reliable screening scales, i.e., there is a need for instruments with high psychometric values for research and compilation of related empirical data [4,5].

Developing skills for solving everyday problems, offering psychosocial support and protective factors at school and in the community, and promoting prevention and health promotion programs are some measures and tasks that society, health institutions, and governments should set as goals. 

The objective of the present study is to develop and evaluate the validity and reliability of an instrument (OTESSED) for the self-detection and prevention of risk factors in eating disorders and related problems (depression, social anxiety, and self-injury) in adolescent samples of boys and girls. It is expected that once the risk areas have been detected, adolescents who so decide may participate in a self-help workshop whose goal is to reduce and prevent the development of these risk factors.

## 2. Materials and Methods

### 2.1. Participants 

The present study corresponds to an instrumental design, with a non-probabilistic sample of N = 577 first-year high school students (277 boys and 300 girls) from Mexican public schools. The age range was between 15 and 19 years. The mean ages of boys and girls were the same, M = 15.61 (SD = 0.73). 

In order for students to be included in the study, they had to meet the following criteria:

- Be in their first year of high school;
- Provide informed consent of their parent or guardian;
- Not have a chronic or disabling medical or psychological problem, and
- voluntary acceptance to participate in the study.

Failure to meet any of these criteria was considered a non-inclusion factor.

### 2.2. Instrument

The Online Test for Self-Screening: Risk Factors of Eating Disorders, Depression, Social Anxiety, and Self-Injury (OTESSED) is a questionnaire that explores the following areas:

Body Weight: Weight and height data are requested to obtain the BMI using the formula weight/height^2^.

Sociodemographic Variables: These 20 items explore data such as age, job, level of studies, and parents’ occupation.

Depression and Suicidal Ideation: The validated version for Mexico was used [14] with 36 items. In addition, four items from the Plutchik [24] Suicide Risk Scale, adapted by Rubio et al. [25], were integrated with multiple-choice answers ranging from “never” to “always”, e.g., I feel that it is not worth continuing in this world; when I am sad, I think about death. 

Body Image Satisfaction and Dissatisfaction [23]: These sections assess satisfaction and dissatisfaction with body image, including positive dissatisfaction with wanting to be thinner and negative dissatisfaction with wanting to be thicker. In addition to having a question that, along with the BMI categories, measures body image disturbance (distorted perception of one’s own body), the responses are classified as overestimation (the person looks thicker than he/she is) and underestimation (looks thinner). Participants are asked to carefully observe nine figures or silhouettes with different body weights and select the one that most resembles or comes closest to their figure. On a second occasion, they are asked to select the one they would most like to have. The difference between these results (how I see myself and how I would like to see myself) provides the scores for satisfaction/dissatisfaction.

Risk Factors Associated with Eating Disorders Scale [21]: This includes 20 items that explore compulsive eating behavior, normal eating behavior, chronic dieting, and feelings of guilt. Likert-type response options ranging from “never” = 1 to “always” = 5 (a higher score implies a more significant problem or risk) were used for the present research. 

Social Anxiety Scale for Adolescents (SAS) [26]: This is a self-report instrument to measure social anxiety. It is composed of three factors: (1) fear of negative evaluation; (2) anxiety and social avoidance in new situations; and (3) anxiety and social avoidance in general. It has 14 questions with Likert-type response options ranging from “never” to “always” (a higher score implies a more important problem or risk).

Self-Injury Scale: This scale [50] has 27 items measuring the frequency, amount, and times of self-injury. For example, have you ever deliberately self-injured or intentionally cut your skin, and how many times have you intentionally cut your skin?

### 2.3. Procedure

The directors of different high school campuses in Mexico City and surrounding areas were asked to invite their students to participate voluntarily in the research. Students who decided to participate were sent the link to the school platform via e-mail. All participants gave informed consent for inclusion before participating in the study. The study was conducted in accordance with the Declaration of Helsinki, and the protocol was approved by the Ethics Committee of the Faculty of Psychology, UNAM (FPCE_ 08032021_H_AC 27/04/2021). This study complied with the General Health Law on Research, considering it is risk-free research; the International Test Commission (ITC) guidelines for online evaluations were followed, as well as those required for UNAM institutional websites.

### 2.4. Statistical Analysis

The Statistical Package for the Social Sciences (SPSS), V.22 (IBM, Mexico city, Mexico) was used. Confirmatory factor analysis (CFA) was applied to the Social Anxiety, Depression, and REB Scales. As a first step, a t-test for independent samples was performed to determine differences by sex. It was established to accept factor loadings > 0.40, with a minimum of three items per factor, KMO ≥ 70, and Bartlett’s sphericity test χ^2^ *p* ≤ 0.05. For the CFA, the AMOS 6.0 program [51] was used, with the maximum likelihood method. The normality of the variables, the absence of extreme values (z ≥ 3), and non-multicollinearity (rxy ≥ 0.90) were tested. The following goodness-of-fit indices were proposed: χ^2^/df ≤ 3; goodness-of-fit index (GFI ≥ 0.90); adjusted goodness-of-fit index (AGFI ≥ 0.90); comparative fit index (CFI ≥ 0.95); Tucker Lewis Index (TLI ≥ 0.90); standardized mean square residual (SMSR ≤ 0.05); root mean square error of approximation (RMSEA ≤ 0.05; 90% CI: 0.00, 0.05) [52,53,54].

The scores obtained with the answers given in the instrument are classified as no risk, low risk, and high risk according to the instrument’s cut-off points based on the instrument’s 25th, 50th, and 75th percentiles [55].

## 3. Results

### 3.1. Sociodemographic Variables

The sociodemographic variables contribute to situate the research problem in the day-to-day social reality and thus to achieve a better understanding of it. For this purpose, information was obtained on important sociodemographic variables related to the objective of this study (Table 1). 

### 3.2. Body Mass Index, Sexual Orientation, and Sexually Active Life

As expected, the highest percentages of girls (72%) and boys (57%) were of normal weight. Twenty-five percent of boys and fourteen percent of girls were overweight, while nine percent of girls and fifteen percent of boys were underweight. Only 4% of boys and 5% of girls fell into the obese category (Table 1). As regards sexual orientation, 72% of girls and 79% of boys responded that they were heterosexual, while 23% of girls reported being bisexual compared to 12% of boys; further, 7% of boys vs 2% of girls reported being homosexual. In terms of sexually active life, 85% of boys vs 93% of girls reported not having initiated their sexual life. Concerning age of first sexual experience, 5% of girls reported being 9 to 11 years old, while boys had their first experience after the age of 11; 32% of boys vs 45% of girls were 12 to 14 years old, and 63% of boys vs 50% of girls were 15 years old or older (Table 1).

### 3.3. Body Image 

Table 1 shows that negative dissatisfaction (desire to be thicker) was found in 50% of boys vs 15% of girls; on the other hand, positive dissatisfaction (desire to be thinner) was found in 68% of girls vs 30% of boys. A perception of negative alteration (underestimation of body weight) was found in 41% of boys vs 29% of girls; on the other hand, a perception of positive alteration (overestimation of body weight) was found in 9% of boys vs 16% of girls.

### 3.4. Physical Activity and Self-Injury

Regarding physical activity, 48% of boys perceived themselves as active people vs 47% of girls, while 52% of boys vs 53% of girls responded as being sedentary. As regards self-injury, 28% of girls responded as having self-injured vs 18% of boys; 17% of boys vs 6% of girls self-injured when they were less than 9 years old; 19% of boys and 25% of girls reported self-injuring from 9 to 11 years old, and 60% of boys and 65% of girls from 12 to 14 years old; 7% of boys and 4% of girls reported self-injuring when they were 15 years old or older (Table 1). 

### 3.5. Construct Validity of the OTESSED 

In order to determine the construct validity, a t-test (independent samples) was performed to determine whether the responses to the different modules of the OTESSED differed by sex. Statistically significant differences were found. Therefore, for each case, it was decided to run a confirmatory factor analysis (CFA) by sex. 

### 3.6. Confirmatory Factor Analysis (CFA) and Models

#### Depression Scale

*Boys.* The results were KMO test = 0.93 and Bartlett’s test of sphericity χ^2^ (78) = 1727.52, *p* ≤ 0.001. Two factors were obtained with 13 items (52% variance explained, overall internal consistency, ϖ = 0.86). F1 with nine items related to feelings of loneliness and depression (29% variance explained, ϖ = 0.88). F2 with four items related to suicidal ideation (23% variance explained, ϖ = 0.82) (Table 2). The cut-off points for the boys’ scale were 16 and 26 for no risk and low risk, respectively, and >27 for high risk.

*Girls.* The results were KMO test = 0.94 and Bartlett’s test of sphericity χ^2^ (55) = 1989.53 *p* ≤ 0.001. There were two factors with 10 items (60% variance explained, ϖ = 0.89). F1 with seven items related to feelings of loneliness and depression (32% of the variance explained, ϖ = 0.88). F2 with three items measured suicidal ideation (28% variance explained, ϖ = 0.89) (Table 2). Table 3 shows the acceptable values of the fit indices for the Depression Scale for both boys and girls. The cut-off points for the girls’ scale were 14 and 23 for no risk and low risk, respectively, and >24 for high risk.

### 3.7. Risky Eating Behavior Scale (REB)

*Boys*. The results were KMO test = 0.77 and Bartlett’s test of sphericity χ^2^ (45) = 818.42, *p* ≤ 0.001. Two factors with nine items were obtained (43% variance explained, overall ϖ = 0.88). F1 with five items measured compulsive eating behavior (27% variance, ϖ = 0.83). F2 with four items measured normal eating behavior (16% variance explained, ϖ = 0.73) (Table 2). Table 3 shows the acceptable values of the fit indices for the REB Scale for both boys and girls. The cut-off points for the boys’ scale were 16 and 26 for no risk and low risk, respectively, and > 27 for high risk. 

*Girls*. The results were KMO test = 0.87 and Bartlett’s test of sphericity χ^2^ (78) = 1950.55, *p* ≤ 0.001. Three factors with 11 items were obtained (56% variance explained, ϖ=.84). F1 with five items measured compulsive eating behavior (25% variance, ϖ = 0.73). F2 with three items measured eating behavior with feelings of guilt (20% variance, ϖ = 0.89). F3 with three items explored normal eating behavior (11% variance explained, ϖ = 0.70) (Table 2). It can also be seen in Table 3 that the fit indices for the Risky Eating Behavior Scale for both sexes (boys and girls) resulted in good values. The cut-off points for the girls’ scale were 18 and 22 for no risk and low risk, respectively, and >27 for high risk.

### 3.8. Social Anxiety Scale

*Boys.* The results were KMO test = 0.90 and Bartlett’s test of sphericity χ^2^ (66) = 1257.74, *p* ≤ 0.001. We obtained two factors with 12 items (58% variance explained, overall ϖ = 0.88). F1 with six items related to social anxiety in new situations (30% variance explained, ϖ = 0.88). F2 with six items measured fear of negative evaluation (28% variance explained, ϖ = 0.88) (Table 2). Table 3 shows the acceptable values of the fit indices for the Social Anxiety Scale for both boys and girls. The cut-off points for the boys’ scale were 16 and 25 for no risk and low risk, respectively, and >26 for high risk.

*Girls*. The results were KMO test = 0.88 and Bartlett’s test of sphericity χ^2^ (91) = 1548.12, *p* ≤ 0.001. Two factors with 11 items were obtained (54% variance explained, overall ϖ = 0.87). F1 with six items related to fear of negative evaluation (31% variance, with ϖ = 0.87). F2 with five items measured social anxiety in new situations (23% variance explained, ϖ = 0.88) (Table 2). As with the other scales, the values of the fit indices of the Social Anxiety Scale (Table 3) were very acceptable, except for the SMSR for boys, which was a little higher than expected The cut-off points for the girls’ scale were 19 and 27 for no risk and low risk, respectively, and >28 for high risk.

### 3.9. Discriminant Validity

Table 4 shows compliance with the criterion that correlations between factors must be less than the square root of the mean variance extracted to meet the discriminant validity.

## 4. Discussion

The aim of the present study focused on the adaptation, psychometric evaluation, validity, and reliability of the OTESSED scales. In addition, this instrument (OTESSED) is used for the self-detection of risk factors and the prevention of eating disorders and related problems (depression, social anxiety, and self-injury) in adolescent samples of males and females.

As expected, the highest percentages of the sample—more than a third of the females and a little more than half of the males—were normal weight (Table 1), and although it can be said that the sample was at risk of obesity (a quarter of the males and an eighth of the females were overweight), in reality, very low percentages of obesity were found (less than five percent) [56,57]. On the other hand, regarding the sexual orientation of the boys/girls in the sample, it was found that more than a third of the boys and girls responded that they were heterosexual. Interestingly, more girls (slightly less than a quarter, almost twice as many as the boys) responded that they were bisexual, while a small percentage of girls (two percent) and boys (seven percent) responded that they were homosexual. These results are striking given that, until recently, the response of young students of being homosexual was second in importance [58], while now, the response of being bisexual appears in second place, leaving homosexual in third place. The appearance of this interesting change in the new student youth requires investigation, given the role of sexuality in this stage of adolescence.

On the other hand, more than four-fifths of the boys and slightly less than the total sample of girls said they had not yet begun their sexual lives. Here, the data raise an interesting research question: if someone, a young man or woman, has not been sexually active, how does he or she know, for example, that he or she is bisexual and not homosexual, or vice versa? Does bisexuality include homosexuality? The answers to these questions require further research.

Concerning body image, results reported in other research were confirmed, showing that more males (half of the sample) than females (half of a third) are dissatisfied with their body image because they wish they had a thicker body with more muscle [32,34]. In contrast, almost two-thirds of females and one-third of males would like to be thinner. The problem with body dissatisfaction is the vulnerability that can lead to developing other problems related to the risk of abnormal eating behavior [23,59]. On the other hand, there was an altered perception of body weight, e.g., more boys (two-fifths) underestimate it than girls (just under one-third). 

In contrast, the overestimation of body weight among boys is minimal (less than ten percent) and slightly less than twice as high among girls. The problem with an altered perception of body weight is its relationship with the maintenance of being overweight and obese. Perceiving oneself as thinner or perceiving one’s child as thinner (when you are not thin) helps little, or not at all, in changing body weight [60,61,62,63].

On the other hand, it is striking that the sample of boys and girls was so homogeneous concerning how they perceived themselves regarding their physical activity or sedentary lifestyle. For example, more than half of the sample rated themselves as sedentary, so less than half considered themselves active [64,65,66]. 

Considering that self-injury is one of the behaviors with the highest health risk (see Table 1), it is worrying that a little more than a quarter of the girls in the sample responded that they have self-injured; boys also responded affirmatively, although in a smaller proportion than girls, confirming this relationship reported in previous research [67,68,69,70]. However, boys start earlier; more boys (slightly more than one-fifth of the sample), almost twice as many as the girls, began self-injury when younger than nine. From this age onwards, the proportion of males and females who self-injure increases, although the percentage of females is always higher. It was found that there is an age peak of higher risk for self-injury between twelve and fourteen years of age, which more than half of the sample indicated in response to the questions on self-injury (Table 2). The information reported thus far is complemented by the data provided by the OTESSED scales.

Three scales were used, each applied to boys and girls: Depression [14,15,16,17,18,19,20,21,22,23,24,25], Social Anxiety [26], and REB (Risky Eating Behavior) [21] (see Table 2 and Table 3), in addition to Body Image [22] (although this has no scale format). The six resulting scales (three for boys and three for girls) showed high values of internal consistency, functionality (easy and quick application due to the small number of items), construct validity (adequate factor loadings and explained variance), and acceptable fit index values (see Table 3). Regarding the Depression Scale, 11 items were obtained for boys and 10 for girls. Both boys and girls presented the same factors: sadness and loneliness, and suicidal ideation, in the same order. As the first factor, sadness and loneliness is the most important (explains the greatest variance), and suicidal ideation is the second factor. For both male and female groups, F1 (loneliness and sadness) is better defined as the first factor, which places it as the most important. While F1 explains the greatest variance, F2, suicidal ideation, shows a difference from F1 since the item “I feel I have no friends or family to count on” is integrated into the male model, showing that for males, the support provided by the family is of great importance.

Concerning the Social Anxiety Scale, two factors were grouped for both boys and girls, unlike the original scale, which grouped three factors for the general population: F1, fear of negative evaluation; F2, anxiety and social avoidance in new situations; and F3, anxiety and social avoidance in general. In the Mexican samples, only two factors were obtained (F1, social anxiety in new situations; F2, fear of negative evaluation). The factors resulted in a different order by sex: for boys, F1 was social anxiety in new situations, which explained the highest percentage of variance, followed by F2, fear of negative evaluation. For girls, F1 was fear of negative evaluation, which explained the highest percentage of variance, followed by F2, social anxiety in new situations. Girls give importance to the opinions of others; in contrast, boys attribute this importance to the confrontation with new social situations. 

In the Risky Eating Behavior (REB) Scale, it was found that girls achieved a more refined categorization of the items than boys; they classified them into three factors (F1, compulsive eating; F2, feelings of guilt; F3, normal eating behavior). On the other hand, boys formed two factors (F1, compulsive eating; F2, normal eating behavior). Interestingly, the most crucial factor was the same for boys and girls, i.e., compulsive eating. In addition, it was found that girls, more than boys, experience high levels of guilt, shame, or self-harm (it was found, as already noted, that girls self-injure more) as well as more frequently using inappropriate coping strategies in the face of emotions perceived as inappropriate [71]. Finally, it is essential to note that the validation process of the instrument was completed, meeting the discriminant validity criterion for each scale of the OTESSED. 

### Strengths and Limitations of the Study

The main strength of this study is that it is a pioneer (in our sociocultural context) in providing a valid and reliable instrument for a population of young people with economic limitations lacking psychological services. This instrument looks for a first approach or recognition of problems related to food, body image, feelings, emotions, and fears (e.g., wondering why self-injury occurs). In this way, the response of seeking and accepting help that otherwise would not be an option due, among other things, to feelings of shame or stigmatization is facilitated.

In the same way, it is important not to overlook the main limitations of this study, such as the non-generalizability of the results of the sample to the general population, given the non-randomization of the population, as well as the preliminary nature of the study that requires further and continuous research to achieve advances, discover new problems, and find new answers. Other limitations include the study’s cross-sectional nature and the self-reporting of variables related to physical activity and sedentary lifestyles. 

Studies on the relationship between depression, social anxiety, and eating disorder-related impairment are scarce worldwide and even more so in emerging countries such as Mexico. This study also seeks to have a valid and reliable instrument as well as a practical or functional one, in the sense of overcoming the problems associated with the traditional practice (face-to-face application); especially for the type of problems we are dealing with (risk factors for eating disorders, social anxiety, depression, and self-harm). As it is known, these problems often cause embarrassment to adolescents, and they tend to avoid asking for help. Therefore, we aim to help the young people in our study to become self-aware and to self-discover the risk factors associated with body image and BMI.

## 5. Conclusions

Based on the above, the following can be concluded: (1) The OTESSED is a valid and reliable instrument at a distance for the self-detection of eating disorder risk factors and related problems (self-injury, social anxiety, and depression) in adolescent samples of males and females. (2) It also has the advantage of being applied remotely, favoring a greater uptake of the adolescent population both because of the ease of responding (online) and the avoidance of the stigma attached to this type of problem (e.g., binge eating, depression, self-injury). For future investigations, we propose a comparison of the present online form of the questionnaire with the corresponding face-to-face version.

## Figures and Tables

**Table 1:** Sociodemographic, body image, physical activity, and self-injury variables.

	Boys	Girls
	n (%)	n (%)
BMI (WHO cut-off points)		
Low weight (<18.5)	40 (14.6%)	26 (8.8%)
Normal weight (18.5–24.9)	159 (56.9%)	219 (72.4%)
Overweight (25–29.9)	68 (24.6%)	41 (13.9%)
Obesity (>30)	10 (4%)	14 (4.8%)
Sexual orientation		
Heterosexual	221 (78.9%)	217 (71.7%)
Bisexual	33 (12.4%)	69 (23.5%)
Homosexual	19 (7.1%)	5 (1.7%)
Other	4 (1.6%)	9 (3.1%)
Sexually active life		
Yes	43 (15.7%)	22 (7.4%)
No	234 (84.3%)	278 (92.6%)
Age of first sexual experience		
9–11 years old	2 (4.9%)	1 (5%)
12–14 years old	13 (31.7%)	9 (45%)
15 years or more	28 (63.4%)	12 (50%)
Body image		
Positive dissatisfaction	84 (30.3%)	204 (68%)
Satisfaction	56 (19.9%)	51 (17%)
Negative dissatisfaction	137 (49.8%)	45 (15%)
Alteration of body image		
Underestimation	113 (41.4%)	85 (28.9%)
No alteration	138 (49.1)	169 (55.4%)
Overestimation	26 (9.5%)	46 (15.7%)
Physical activity		
Activity (e.g., you walk, run, move)	132 (47.7%)	141 (47%)
Sedentary (e.g., you spend most of your time sitting down)	145 (52.3%)	159 (53%)
Self-injury		
Yes	49 (17.7%)	84 (28%)
No	228 (82.3%)	216 (72%)
Age at first self-injury		
Less than 9 years old	6 (14%)	5 (6.2%)
9–11 years old	8 (18.6%)	20 (24.7%)
12–14 years old	26 (60.5%)	56 (65.4%)
15 years or more	2 (6.9%)	3 (3.7%)
Types of injuries		
Skin injuries (cuts, burns, etc.)	19 (38%)	20 (23.8%)
Blows (head, broken bones)	3 (3.9%)	13 (15.5%)
Suicide attempts	12 (24.2%)	51 (60.7%)
I prefer not to say	15 (33.9%)	

**Table 2:** Depression, REB, and Social Anxiety models for boys and girls.

Factor	Models	Load Factor	ϖ	ϖ Total
Depression				
F1 Boys	I feel be happy anywhere	0.77	0.88	0.86
	I feel tired and indifferent	0.75		
	I have felt useless and worthless	0.69		
	I feel so lonely that I cannot tolerate it	0.72		
	I think that no one misses me if I am not there	0.72		
	I feel isolated from people	0.68		
	I feel that people do not accept me and what I do	0.69		
F2 Boys	I have commented on occasion that I would like to disappear from this world	0.72	0.82	
	I feel that it is not worthwhile to continue in this world	0.71		
	When I am sad, I think of death	0.75		
	I feel I have no friends or family I can count on	0.75		
F1 Girls	I feel so lonely that I cannot tolerate it	0.72	0.88	0.89
	I feel isolated from people	0.67		
	I feel that people do not accept me and what I do	0.70		
	I think that no one misses me if I am not there	0.76		
	I feel I can’t be happy anywhere	0.79		
	I feel tired and indifferent	0.77		
	I feel I have no friends or family I can count on	0.70		
F2 Girls	I feel that it is not worthwhile to continue in this world	0.88	0.89	
	When I am sad, I think about death	0.87		
	I have commented on occasion that I would like to disappear from this world	0.83		
**Risky Eating Behavior**				
F1 Boys	I feel I can’t stop eating	0.72	0.83	0.88
	My problem is to start eating, but when I start, I can hardly stop	0.65		
	I eat large amounts of food even when I am not hungry	0.74		
	I eat as without measure	0.75		
	I feel that I eat more than most of my friends do	0.58		
F2 Boys	I make sure that my food is varied (contains the food groups of the “good food plate”)	0.97	0.73	
	I want my diet to be nutritious	0.47		
	I drink natural water (at least 3 glasses a day)	0.48		
	I eat three meals and two snacks (as indicated in the plate of good food)	0.53		
F1 Girls	I eat as without measure	0.83	0.73	0.84
	I feel I can´t stop eating	0.85		
	I eat large amounts of food even when I am not hungry	0.78		
	I feel that I eat more than most of my friends do	0.69		
	My problem is to start eating, but when I start I can hardly stop	0.84		
F2 Girls	I get depressed when I overeat	0.84	0.89	
	I think that the problems, instead of taking away my appetite, increase it	0.78		
	I’m ashamed to eat so much	0.76		
F3 Girls	I make sure that my food is varied (contains the food groups of the “good food plate”)	0.91	0.70	
	I eat three meals and two snacks (as indicated in the plate of good food)	0.62		
	I want my diet to be nutritious	0.42		
**Social Anxiety**				
F1 Boys	I worry about what others say about me	0.86	0.88	0.88
	I worry about not being liked by others	0.86		
	I worry about what others think of me	0.74		
	I worry about being evaluated by others	0.83		
	I think that what I do (the way I act) may not be liked by others	0.64		
	When I ask others to do something for me (e.g., a favor) I am afraid that they will say no.	0.55		
F2 Boys	When I talk to people I don’t know well, I get nervous, even if they are my age	0.81	0.88	
	I get nervous when I am with other people	0.79		
	When I am in a group, I keep quiet	0.76		
	I feel embarrassed even when I am with people I know well	0.73		
	When I am surrounded by strangers, I am embarrassed to be seen, move or talk	0.75		
	I only talk to people I know well	0.61		
F1 Girls	I worry about what others say about me	0.87	0.87	0.87
	I worry about what others think of me	0.80		
	I worry about not being liked by others	0.80		
	I worry about being evaluated by others	0.75		
	When I ask others to do something for me (e.g., a favor) I am afraid that they will say no	0.66		
	I think that what I do (the way I act) may not be liked by others	0.59		
F2 Girls	I get nervous when I am introduced to strangers	0.66	0.88	
	When I talk to people I don’t know well, I get nervous, even if they are my age	0.69		
	I get nervous when I am with other people	0.80		
	When I am surrounded by strangers, I am embarrassed to be seen, move or talk	0.75		
	When I am in a group, I keep quiet	0.57		

**Table 3:** Index of goodness of fit.

Scale	Sex	X^2^	Sig	RMSEA	CFI	TLI	SMSR
Depression	Boys	2	0.000	0.05	0.97	0.96	0.03
	Girls	2.3	0.000	0.05	0.97	0.96	0.05
REB	Boys	2.2	0.000	0.06	0.96	0.95	0.06
	Girls	2	0.000	0.06	0.97	0.95	0.06
Social Anxiety	Boys	2	0.000	0.07	0.95	0.94	0.08
	Girls	2.5	0.000	0.07	0.95	0.94	0.06

Note: Root mean square error of approximation (RMSEA); Comparative fit index (CFI); Standardized mean square residual (SMSR); Tucker Lewis Index (TLI).

**Table 4:** Correlation between variables and average variances.

	Factors	AVE	F1	F2	F3
Depression, Boys	F1	0.52	0.72		
	F2	0.54	0.07 *	0.73	
Depression, Girls	F1	0.53	0.73		
	F2	0.74	0.65 *	0.86	
Risky Eating Behaviors, Boys	F1	0.47	0.69		
	F2	0.46	0.07 *	0.68	
Risky Eating Behaviors, Girls	F1	0.64	0.80		
	F2	0.63	0.51 *	0.79	
	F3	0.50	0.03 *	0.02 *	0.70
Social Anxiety, Boys	F1	0.57	0.75		
	F2	0.55	0.31 *	0.74	
Social Anxiety, Girls	F1	0.56	0.75		
	F2	0.50	0.29 *	0.70	

* Note: All correlations are significant (*p* < 0.01). The square root of the AVE is shown on the diagonal.
